# Variations in Nitrogen Metabolism are Closely Linked with Nitrogen Uptake and Utilization Efficiency in Cotton Genotypes under Various Nitrogen Supplies

**DOI:** 10.3390/plants9020250

**Published:** 2020-02-15

**Authors:** Asif Iqbal, Qiang Dong, Xiangru Wang, Huiping Gui, Hengheng Zhang, Xiling Zhang, Meizhen Song

**Affiliations:** State Key Laboratory of Cotton Biology, Institute of Cotton Research of Chinese Academy of Agricultural Sciences, Anyang 455000, China; asif173aup@gmail.com (A.I.); dongqiang@caas.cn (Q.D.);

**Keywords:** cotton, contrasting genotypes, key-traits, nitrogen use efficiency, nitrogen metabolism

## Abstract

Cotton production is highly sensitive to nitrogen (N) fertilization, whose excessive use is responsible for human and environmental problems. Lowering N supply together with the selection of N-efficient genotypes, more able to uptake, utilize, and remobilize the available N, could be a challenge to maintain high cotton production sustainably. The current study aimed to explore the intraspecific variation among four cotton genotypes in response to various N supplies, in order to identify the most distinct N-efficient genotypes and their nitrogen use efficiency (NUE)-related traits in hydroponic culture. On the basis of shoot dry matter, CCRI-69 and XLZ-30 were identified as N-efficient and N-inefficient genotypes, respectively, and these results were confirmed by their contrasting N metabolism, uptake (NUpE), and utilization efficiency (NUtE). Overall, our results indicated the key role of shoot glutamine synthetase (GS) and root total soluble protein in NUtE. Conversely, tissue N concentration and N-metabolizing enzymes were considered as the key traits in conferring high NUpE. The remobilization of N from the shoot to roots by high shoot GS activity may be a strategy to enhance root total soluble protein, which improves root growth for N uptake and NUE. In future, multi-omics studies will be employed to focus on the key genes and pathways involved in N metabolism and their role in improving NUE.

## 1. Introduction

Nitrogen (N) is one of the most important and limiting nutrients for crop production [1]. It is the main component of several macromolecules, which are necessary for normal growth and productivity [2]. N fertilization has significantly improved crop production and thus reduces the pressure of global population growth [1]. However, low N is a major constraint in crop production and can decrease the yield up to 50% [3,4]. To maintain better crop growth and production, the demand for N fertilizer increases [5,6] and this increase may continue up to threefold in the future [7]. On the other side, plants can only utilize half of the applied fertilizer [8], and the rest of fertilizer leaks into groundwater and causes a great threat in terms of human and environmental problems [9,10]. Therefore, it is an urgent need to enhance N use efficiency (NUE) in plants to reduce N fertilizer application into soils.

The intraspecific variation in the genotypes belongs to the same species are the valuable resources for breeding the desirable traits for further improvement [11]. The use of crop genotypes to select the ideal form with desirable traits is an effective approach in order to attain high NUE [12]. Significant progress has been made in Arabidopsis, and different accessions have been used for exploiting natural variations in N uptake, utilization, and NUE in response to various N supply [12,13]. Moreover, such approaches also have been applied in crop plants such as rice [14], wheat [15], maize [16], rapeseed [17], tomato [18,19], soybean [20], and barley [21]. In all these studies, the main focus was to observe the natural variations in terms of selecting plants with high NUE. Besides these crops, very little attention has been paid to understanding the intraspecific variation in cotton genotypes for NUE.

NUE is a complex trait affected by both genetic and environmental factors [22]. It is composed of two main components, N uptake efficiency (NUpE) and N utilization efficiency (NUtE), which are influenced by biochemistry, phenology, and architecture, as well as the external environment such as N availability [23,24]. Studies have shown the role of total plant dry matter, nitrogen absorption, and root morphological traits in improving NUE [19,25,26]. However, few studies have been conducted to understand the role of N metabolism [27]; specifically, the enzymes involved in N metabolism such as nitrate reductase (NR), glutamine synthetase (GS), glutamate synthase (GOGAT), and glutamate dehydrogenase (GDH), which play central roles in the primary assimilation of N [28,29]. In addition, the N-containing compounds such as amino acids and proteins are also used as indicators to detect genotypic responses to the N supply [21]. These N-containing compounds regulate N uptake, assimilation, and metabolism in the plants on the basis of external N availability. Therefore, the key enzymes involved in N assimilation and the final products of N metabolism are the most important biochemical features for improving NUE [6]. Thus, investigating the levels of these indicators in cotton with contrasting N-efficient genotypes will increase our understanding of N metabolism and may be useful in identifying and improving NUE in response to various nitrogen supplies.

Cotton (*Gossypium* L.) is grown all over the world and is the backbone of the world’s natural textile fibers [30]. Among the production inputs, high N fertilization is a big challenge that not only increases the cost for farmers but also brings environmental and human problems [31]. To reduce this costly component of crop production, there is an immediate need to identify and develop cotton genotypes with high NUEs. In the current study, four contrasting N-efficient cotton genotypes were exposed to various nitrogen levels for 4 weeks to investigate the variation in NUE-related traits such as shoot dry matter, NUpE, NUtE, and N metabolism. Global ANOVA was conducted to find the relative contribution of N supply, genotype, and their interaction for each studied trait. Similarly, principal component analysis (PCA) was performed to understand the N response patterns of contrasting N-efficient cotton genotypes and the most essential traits contributing to NUE. Furthermore, correlation analysis was also performed to know the possible correlations among all the studied traits that might explain the variations in NUpE and NUtE among the genotypes under various N supply. Consequently, the objectives of our study were (1) to investigate the interspecific variation for NUE-related traits in cotton genotypes and (2) to find out the most distinct genotypes for contrasting NUE in response to various nitrogen supplies.

## 2. Results

### 2.1. Growth and Photosynthesis

The results of the current study showed a dramatic reduction in shoot length (67%), total plant dry matter (44%), and single leaf area (83%) under low nitrogen (0.25 mM) as compared to high nitrogen levels. However, the reduction was more prominent for N-inefficient genotypes compared to N-efficient genotypes (Table 1). The shortest shoot length (30%), least total dry matter (25%), and the smallest single leaf area (40%) were from genotype XLZ-30, whereas the greatest values were from genotype CCRI-69 (Table 1).

The leaf photosynthetic traits such as net photosynthetic rate, stomatal conductance, transpiration rate, and intercellular CO_2_ concentration of cotton genotypes were differentially influenced by various nitrogen supplies (Table 1). The results showed that high nitrogen significantly increased photosynthetic rate (32%), stomatal conductance (6%), and transpiration rate (24%), whereas intercellular CO_2_ concentration was 46% lower as compared to low nitrogen supply (Table 1). In particular, CCRI-69 had the highest photosynthetic rate (17%), stomatal conductance (7%), and transpiration rate (16%), whereas XLZ-30 had the lowest values for these three traits at each nitrogen level (Table 1). The high intercellular CO_2_ concentration level under low nitrogen level (0.25 mM), especially in N-inefficient genotypes, indicated their lower efficiencies in carboxylating the available carbon dioxide (Table 1).

### 2.2. Nitrogen Use Efficiency

The performance of genotypes was analyzed by directly comparing the values of α (shoot dry matter) and β (nitrogen levels) using non-linear regression analysis [19] (Table 2). The values for α or maximum shoot dry matter ranged from 1.99 to 2.79 g for XLZ-30 and CCRI-69, which had the lowest and highest values among all the genotypes, respectively (Table 2). The values of β or nitrogen level used to produce maximum shoot dry matter ranged from 0.24 to 0.59 for CCRI-69 and XLZ-30, respectively, showing the lowest and highest values among the cotton genotypes. The results suggested that N-inefficient genotypes, particularly XLZ-30, needed significantly more nitrogen to achieve their maximum shoot dry matter than the N-efficient genotype CCRI-69 (Table 2). Thus, the genotypes CCRI-69 and XLZ-30 exhibited contrasting responses (α and β) and were considered the most distinct N-efficient and N-inefficient genotypes, respectively (Table 2).

**Figure 1:**
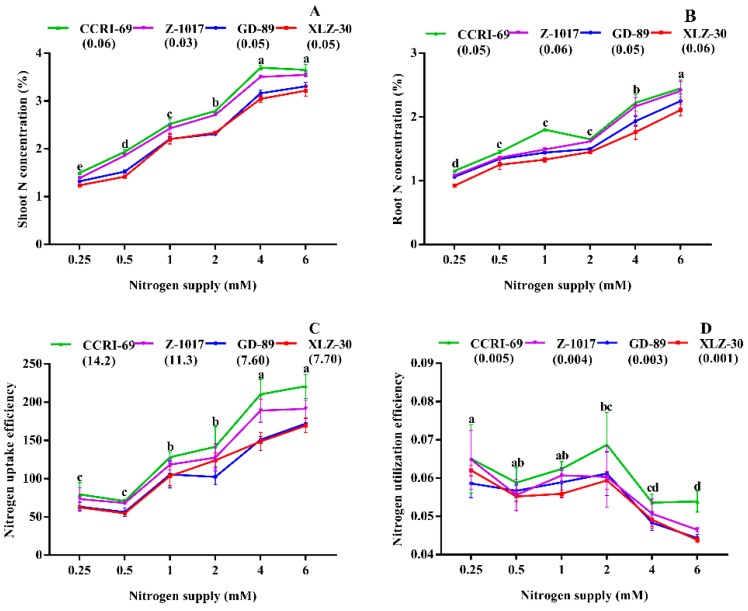
(**A**) Shoot nitrogen concentration (%), (**B**) root nitrogen concentration (%), (**C**) N uptake efficiency (mg N g^−1^ RDW), and (**D**) N utilization efficiency (NUtE, g^2^ RDW mg^−1^) of contrasting nitrogen-efficient cotton genotypes under various nitrogen concentrations (0.25, 0.5, 1, 2, 4, or 6 mM NO_3_^−^). The values are presented as mean ± standard error (*n* = 9).

A significant difference was noted for the shoot and root N concentrations in response to N supply (Figure 1A,B). The N concentration in the shoot and root of cotton genotypes significantly decreased by 51%–60% and 41%–54% under 0.5 and 0.25 mM nitrogen, respectively. In CCRI-69, N concentration in the shoot and root were enhanced by 16% and 18%, respectively compared to XLZ-30 (Figure 1A,B). No significant difference was observed in shoot N concentration at high N levels (4–6 mM) and moderate N levels (1–2 mM) in the root (Figure 1A,B).

Nitrogen uptake efficiency was significantly improved by 63%–67% under high nitrogen 6 mM as compared to 0.5 mM and 0.25 mM nitrogen. However, the increase was more obvious for CCRI-69 compared with other genotypes, especially GD-89 and XLZ-30, which showed a reduction of 22% and 24%, respectively (Figure 1C). Conversely, nitrogen utilization efficiency decreased with an increase in nitrogen supply; however, low nitrogen levels improved nitrogen utilization efficiency by 20%–33% (Figure 1D). The increase in nitrogen utilization efficiency was 10% higher in CCRI-69 as compared to XLZ-30 (Figure 1D). To better understand these genotypic differences, the activities of enzymes involved in N metabolism were also determined.

### 2.3. Nitrogen Metabolism

**Figure 2:**
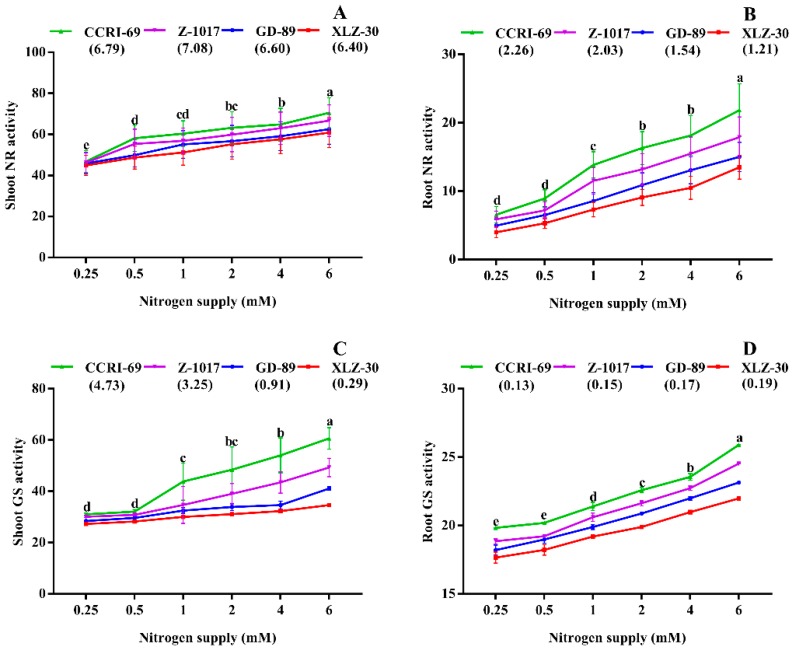
(**A**) Shoot nitrate reductase activity (µg g^−1^ FW h^−1^), (**B**) root nitrate reductase activity (µg g^−1^ FW h^−1^), (**C**) shoot glutamine synthetase activity (µmol g^−1^ FW h^−1^), (**D**) and root glutamine synthetase activity (µmol g^−1^ FW h^−1^) of contrasting nitrogen efficient cotton genotypes under various nitrogen concentrations (0.25, 0.5, 1, 2, 4, or 6 mM NO_3_^−^). The values are presented as mean ± standard error (*n* = 9).

To understand the genotypic difference in nitrogen metabolism, we monitored the activities of enzymes involved in N metabolism. The results showed that both shoot and root NR activities were increased with an increase in nitrogen supply. Under high nitrogen supply, shoot and root NR activity increased by 29% and 69% as compared to low nitrogen supply, respectively (Figure 2A). Similarly, shoot and root NR activities of CCRI-69 increased by 12% and 42% as compared to XLZ-30, respectively (Figure 2A). Both shoot and root GS activities were increased with an increase in nitrogen supply (Figure 2C,D). However, both shoot and root GS activities were larger in CCRI-69 compared to XLZ-30. The activity state of GS in shoot and root were significantly different at 32% and 13% for CCRI-69 and XLZ-30, respectively (Figure 2C,D).

**Figure 3:**
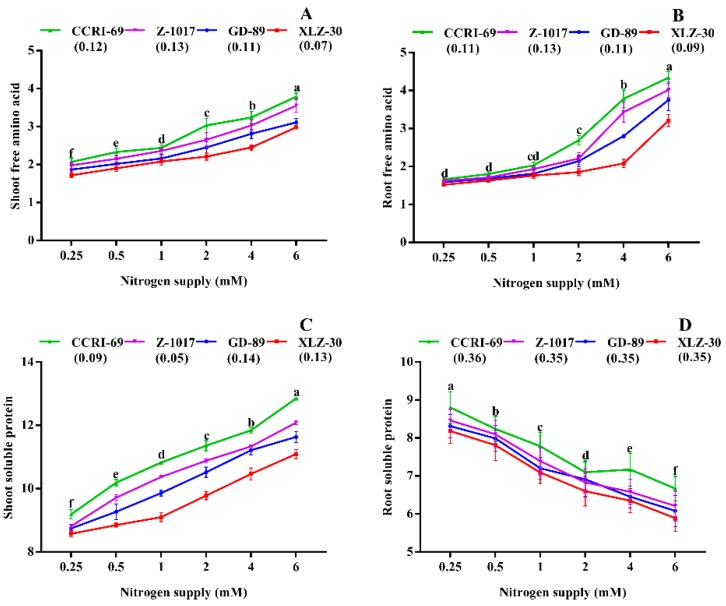
(**A**) Shoot glutamate synthase activity (shoot glutamate synthase (GOGAT), U mg^−1^ protein), (**B**) root glutamate synthase activity (root GOGAT, U mg^−1^ protein), (**C**) shoot glutamate dehydrogenase (shoot glutamate dehydrogenase (GDH), U mg^−1^ protein), and (**D**) root glutamate dehydrogenase activity (root GOGAT, U mg^−1^ protein) of contrasting nitrogen efficient cotton genotypes under various nitrogen concentrations (0.25, 0.5, 1, 2, 4, or 6 mM NO_3_^−^). The values are presented as mean ± standard error (*n* = 9).

Nitrogen supply had a significant influence on GOGAT and GDH activities, which tended to increase with an increase with nitrogen levels (Figure 3). The increase in shoot and root GOGAT activities of CCRI-69 were 32% and 21%, respectively, as compared to XLZ-30 (Figure 3A,B). Similarly, shoot (14%) and root (19%) GDH activities were significantly increased in CCRI-69 compared with XLZ-30 (Figure 3C,D).

**Figure 4:**
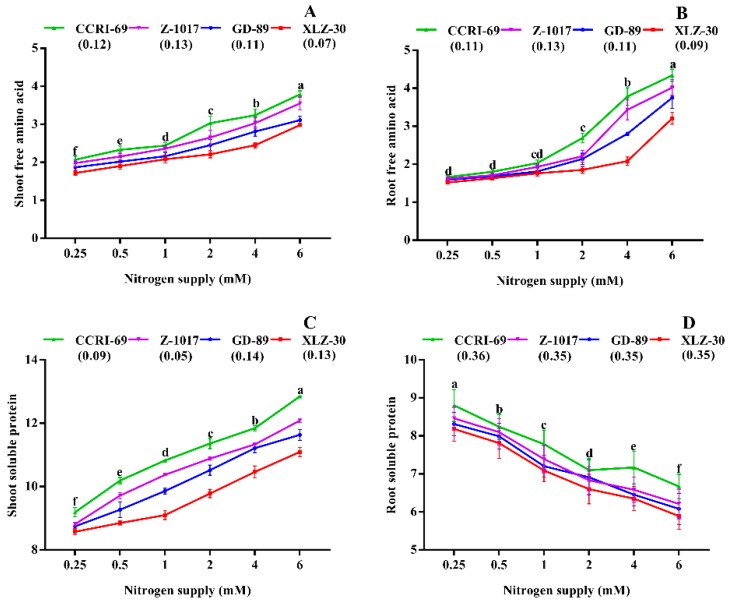
(**A**) Shoot free amino acid (mg g^−1^), (**B**) root free amino acid (mg g^−1^), (**C**) shoot-soluble protein (mg g^−1^), and (**D**) root-soluble protein (mg g^−1^) of contrasting nitrogen-efficient cotton genotypes under various nitrogen concentrations (0.25, 0.5, 1, 2, 4, or 6 mM NO_3_^−^). The values are presented as mean ± standard error (*n* = 9).

The final products of nitrogen metabolism such as amino acids and total soluble proteins were also significantly affected by nitrogen supply (Figure 4). A significant increase in shoot and root free amino acid was observed under high nitrogen supply (Figure 4A,B). However, the increase in both shoot and root free amino acid in CCRI-69 was significantly higher than XLZ-30 (Figure 4A,B). Consistently, shoot-soluble protein was increased by 26% under high nitrogen. In contrast, root-soluble protein was reduced by 36% under high nitrogen supply. Both shoot and root total soluble protein in CCRI-69 were 13% and 8% higher than XLZ-30, respectively (Figure 4C,D).

### 2.4. ANOVA and Principal Component Analysis

**Figure 5:**
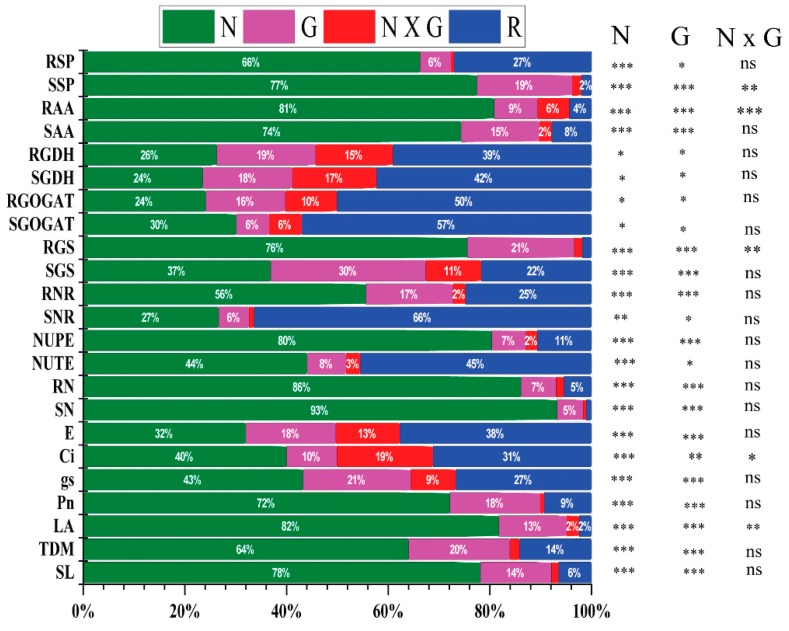
ANOVA of morphological, physiological, and biochemical traits of contrasting nitrogen-efficient cotton genotypes in response to various nitrogen concentrations (0.25, 0.5, 1, 2, 4, and 6 mM). The main effects (nitrogen (N) and genotypes (G), interaction (N × G)) and residual (R) are represented as percentage of type III sums of squares. *p*-values of the *F*-test are indicated. * *p* < 0.05; ** *p* < 0.01; *** *p* < 0.001; ns: not significant. RSP, root-soluble protein; SSP, shoot-soluble protein; RAA, root-free amino acid; SAA, shoot-free amino acid; RGDH, root GDH activity; SGDH, shoot GDH activity; RGOGAT, root GOGAT activity; SGOGAT, shoot GOGAT activity; RGS, root GS activity; SGS, shoot GS activity; RNR, root NR activity; SNR, shoot NR activity; NUpE, N uptake efficiency; NUtE, N utilization efficiency; RN, root N concentration; SN, shoot N concentration; E, transpiration rate; Ci, intercellular CO_2_ concentration; gs, stomatal conductance; Pn, net photosynthesis; LA, leaf area, TDM, total plant dry matter; SL, shoot length.

In the current study, 23 different morphophysiological and biochemical traits were analyzed, which had significant effects for nitrogen supply (N), genotypes (G), and their interaction (N × G). N supply significantly affected the detected traits and described 24%–93% of the total variance. For some traits (shoot N concentration, root N concentration, single leaf area, root amino acid, N uptake efficiency, shoot length, shoot-soluble protein, root glutamine synthetase, shoot free amino acid, net photosynthetic rate, root-soluble protein, total plant dry matter, and root nitrate reductase activity), the effects of nitrogen supply accounted over 50% of the total variance (Figure 5). The genotypic effects were also significant for all the traits and described 5%–30% of the total variance. The maximum genotypic difference was noted for shoot glutamine synthetase activity (Figure 5). Interaction effects also had a vital role in total variance, and about eight traits (root free amino acid, shoot-soluble protein, root glutamine synthetase, leaf area, and intercellular CO_2_ concentration) had significant effects and contributed 2%–19% of the total variance. Among the detected traits, the highest interaction effect was noted for intercellular CO_2_ concentration, which explained 19% of the total variance (Figure 5).

**Figure 6:**
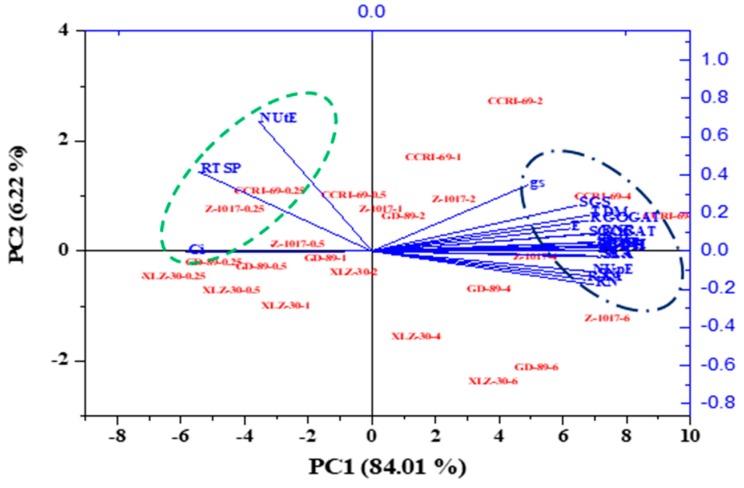
Principal component analysis (PCA) of morphophysiological and biochemical traits of contrasting N-efficient cotton genotypes in response to various nitrogen concentrations (0.25, 0.5, 1, 2, 4, and 6 mM NO_3_^−^). The PCA shows the biplot of the first two principal components. The eigenvectors are shown in Table S1.

The principal component analysis was also performed to disclose the response patterns of cotton genotypes to N supply and the key traits contributing to NUE. The loading plots of principal components 1 and 2 of the PCA analysis included 23 selected traits obtained from the average values of the four genotypes under various nitrogen supplies (Figure 6 and Table S1). Nitrogen levels were associated with PC1 and explained 84.01% of the variation. Cotton genotypes were associated with PC2 and contributed 6.22% of the total variation. Growth, photosynthetic rate, tissue N concentrations, N-metabolizing enzymes, and NUpE were the key contributors to PC1, whereas stomatal conductance, shoot GS activity, root total soluble protein, and NUtE were the key contributors to PC2 (Figure 6 and Table S1). Thus, the results suggested that the genotypes had a large variation for NUtE, which was also shown in the ANOVA.

### 2.5. Correlation Analysis

**Figure 7:**
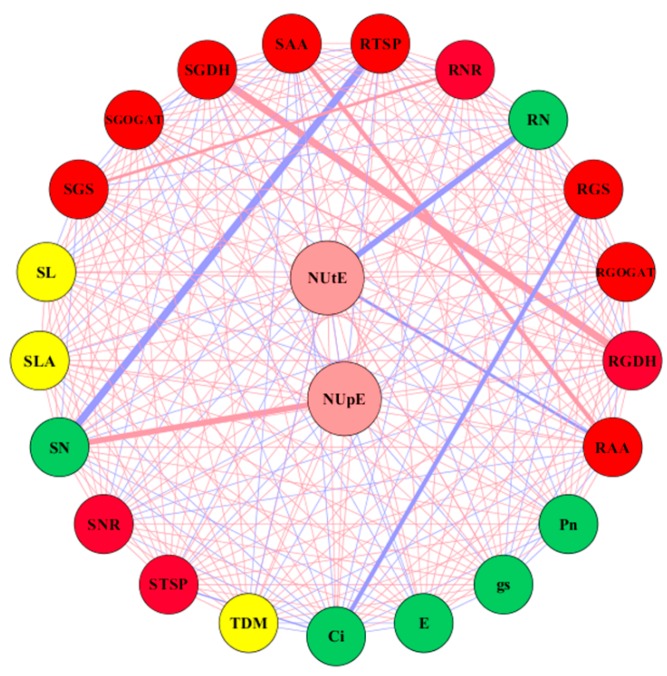
Relationships between morphological (**yellow**), physiological (**green**), and biochemical (**red**) traits with NUpE and NUtE (**brown**). Nodes represent the traits and edges (**red**, positive; **blue**, negative) represent the correlations. The thickness of the edges represents the strength of correlation coefficient for each pair. TDM, total plant dry matter; SL, shoot length; SLA, single leaf area; Pn, net photosynthesis; gs, stomatal conductance; E, transpiration rate; Ci, intercellular CO_2_ concentration; SN, shoot nitrogen concentration; RN, root nitrogen concentration; SNR, shoot nitrate reductase activity; RNR, root nitrate reductase activity; SGS, shoot glutamine synthetase activity; RGS, root glutamine synthetase activity; SGOGAT, shoot glutamate synthase activity; RGOGAT, root glutamate synthase activity; SGDH, shoot glutamate dehydrogenase activity; RGDH, root glutamate dehydrogenase activity; SAA, shoot-free amino acid; RAA, root-free amino acid; STSP, shoot total soluble protein; RTSP, root total soluble protein.

To know the key traits contributing to nitrogen uptake and utilization efficiency, we selected 3 morphological, 6 physiological, and 12 biochemical traits for Pearson correlation analysis. The visualization in Cytoscape revealed that a total of 21 nodes (traits) were connected in the network with 253 edges in the network (Figure 7). Out of the total direct correlation, 15 traits had positive correlations and 7 traits had negative correlations with nitrogen uptake efficiency (Figure 7). Among the traits, shoot N concentration had a strong positive correlation (*r* = 0.86) with nitrogen uptake efficiency. Nitrogen utilization efficiency had a positive correlation with 11 traits and also a negative correlation with 11 traits. Among the traits, root total soluble protein and shoot GS activity had a strong positive relationship with NUtE (Figure 7).

## 3. Discussion

### 3.1. Variations in Morphophysiological and Biochemical Traits among Cotton Genotypes

It has been observed that most of the plants showed some behavioral changes in response to changing environments [32] such as N availability [33]. This resulted in a decrease in growth, photosynthetic activity, translocation of N, and accumulation of pigments [34]. These different responses of plants help the researchers to select the potential genotypes. Previous studies showed a significant variation in the *Arabidopsis* [35] and crops such as rapeseed [36], maize [37], and barley [21] under contrasting nitrogen supply, and as a result significant genotypic variation was found in the traits contributing to NUE [36]. In cotton, very few studies have been performed to understand the key traits contributing to NUE in different genotypes under contrasting nitrogen supply. In the current study, large phenotypic differences were found among the cotton genotypes. Relatively higher morphological traits such as shoot length, shoot dry matter, total plant dry matter, and single leaf area were noted in N-efficient genotypes (CCRI-69 and Z-1017) as compared to N-inefficient genotypes (XLZ-30 and GD-89). The increase in these morphological traits in N-efficient genotypes might be due to improve N uptake, photosynthetic activity, and N metabolism. The current results are consistent with the previous studies performed on *Arabidopsis* [11], maize [38], tomato [19], and cotton [39], where efficient genotypes performed better than inefficient genotypes. In addition, ANOVA showed that the genotypic effects accounted for 5%–30% of the total variance, suggesting that cotton genotypes have significant variations in morphological traits. In PCA analysis, PC2 clearly determined that the genotypic effect and total plant dry matter contributed more to the genotypic effect. Thus, cotton genotypes showed considerable variation in morphological traits under various nitrogen supplies.

In addition to morphological traits, a clear difference among the genotypes was observed for photosynthetic traits. Photosynthesis is very sensitive to N availability [40,41] because 57% of the leaf nitrogen is located in the chloroplast and is used for the synthesis of photosynthetic components and related enzymes [6,42]. The previous study showed a positive correlation between leaf N concentration and photosynthesis [43,44] and, consistently, a positive correlation was noted between N concentration and photosynthetic activity (*r* = 0.93). Similarly, the increasing trend of photosynthesis with N supply in N-efficient genotype (CCRI-69) was consistent with our previous results [39], as well as results in poplar species [45,46]. Conversely, the photosynthetic activity in N-inefficient genotypes especially XLZ-30 decreased, which might have been due to inhibition in the photosystem, as many genes involved in the photosystem were downregulated under contrasting nitrogen supply [46]. Similarly, low nitrogen significantly decreased the photosynthetic activities in cotton genotypes, which are consistent with the previous results in which *Arabidopsis*, rice, maize, wheat, and other nitrogen-deficient plants were reduced [47,48,49,50]. This reduction in photosynthetic activities under low nitrogen may also be due to poor carboxylation, as noted from the high levels of intercellular CO_2_ concentration, which are in line with the results obtained in rice and sunflower [51]. Thus, these results suggested that N is important for photosynthesis and the genotypes having high photosynthetic activity can increase growth, productivity, and NUE.

To keep normal growth and productivity under low N supply, improvement in NUE is very important [6,7]. Subsequently, identifying the potential genotypes under various N supply is the best way to understand the mechanism and the key traits contributing to NUE [52]. There are many methods to measure NUE; however, we have compared cotton genotypes by shoot dry matter and its relation to nitrogen supply, which was previously described for the identification of efficient genotypes in legumes [53] and tomato [19]. In the current study, a large genotypic difference was found in shoot dry matter and the amounts of nitrogen used to achieve the maximum shoot dry matter (Table 2). Conclusively, CCRI-69 and XLZ-30 were considered the most distinct N-efficient and N-inefficient genotypes, respectively. Moreover, the contrasting behavior of these genotypes was confirmed from N uptake, utilization, and metabolism levels. The lower potential of XLZ-30 might be due to low photosynthetic efficiency, poor N uptake, utilization, and metabolism as described in the Results section, and these results are consistent with the previous studies [54,55].

In addition, the enzymes involved in N metabolism were measured to find the potential of genotypes for N metabolism. After uptake, nitrogen reduced in the roots or is translocated to the shoots for assimilation [6]. During primary assimilation, nitrogen is converted into glutamine and glutamate following the synthesis of amino acids, proteins, and other nitrogenous compounds [56]. This primary assimilation of nitrogen is accompanied by various enzymatic activities such as NR, GS, GOGAT, and GDH [33,57]. In the current study, large genotypic variations were observed among the key enzymes regulating N metabolism. Interestingly, enzymatic activities in CCRI-69 were the highest among the studied genotypes, indicating its greater potential for N metabolism (Figure 2 and Figure 3). In consistent with the current results, a significant increase in N enzymatic activities were found in [58] and N-efficient *Brassica napus* genotypes [59]. This genotypic difference in enzymatic activities might be due to differences in transporters and N fluxes in the roots [60]. This assumption is based on the Km data of rice and *Arabidopsis* nitrate transporters, as described in a previous study [61]. In line with these results, the α value, which is similar to Km, was higher in CCRI-69, suggesting that the activities of cotton transporters may be similar to those of rice transporters. Thus, we assumed that in CCRI-69, the low-affinity transporters may be more active in the roots and shoots, which might lead to the high N uptake and assimilation even under low nitrogen supply, as discussed in previous studies [61,62]. Another reason for high N metabolism in CCRI-69 might be due to the constant conversion of nitrate to nitrite and then to ammonia and amino acids [63,64]. Thus, a well-coordinated system of N uptake and assimilation may be the base of the high N use efficiency in CCRI-69 (Yun et al., 2008), which was explained by their high enzymatic activities (Figure 2 and Figure 3).

In general, the efficiency of any process can be verified from the end products. The final products of N metabolism are amino acids and proteins [65,66]. Therefore, free amino acids and total soluble protein were also measured to further find the genotypic difference in N metabolism. As discussed in the Results section, nitrogen-efficient genotypes have significantly more soluble protein, free amino acids, and soluble sugar than inefficient genotypes. Under low nitrogen, a significant increase in the root total soluble protein and total soluble sugar were observed in the cotton genotypes. As mentioned in the Results section, a significant reduction in free amino acids and shoot total soluble proteins were observed under low nitrogen supply, whereas root total soluble protein was increased under low nitrogen supply (Figure 4A–C). The increase in root total soluble protein was more prominent in CCRI-69, which is in line with the results obtained in maize ear [67]. The reason for this high root-soluble protein might be due to an increase in transcript levels encoding various proteins [68], or due to higher protease activity in the roots, resulting in a greater level of protein degradation [69]. Moreover, this increase in root total soluble protein acting as N sources for photosynthetic processes and CCRI-69 can transfer more N to shoots and, in return, more carbon to roots, resulting in better growth and NUE. Thus, clear genotypic differences in cotton were noted for N metabolism and its importance in increasing NUE.

### 3.2. Variations in Morphophysiological and Biochemical Traits Are Associated with NUE

ANOVA and principal component analysis showed that most of the traits were affected by nitrogen supply and contributed to the total variation explain by PC1. Shoot glutamine synthetase activity and NUtE shared most of the genotypic variation and contributed to PC2, which was mainly associated with cotton genotypes (Figure 5 and Figure 6). Moreover, NUtE was negatively correlated with increasing nitrogen supply, which is in line with the previous results [70,71,72]. The reduction in NUtE with increasing nitrogen supply might be due to excessive storage of external N beyond plant requirements, and thus the external N cannot stimulate further growth [73]. As a result, lower growth, with a high tissue N concentration, may reduce NUtE under high N supply.

To uncover the relationship between NUpE and NUtE with morphophysiological and biochemical traits, a correlation network was constructed (Figure 7). Interestingly, NUpE had direct interaction with 22 traits, having 15 negative and 7 positive correlations. The N concentration in the shoot and N-metabolizing enzymes were strongly associated with NUpE and shared a large N effect, which indicated that N uptake and metabolism were more affected by nitrogen supply than genotype. Thus, N-metabolizing enzymes may be targets for increasing NUE. Similarly, NUtE had a strong positive correlation with root total soluble protein and shoot GS activity (Figure 7). Moreover, root total soluble protein and NUtE were higher under low nitrogen supply, which is consistent with the results of the previous studies [45,74]. This increase in NUtE and root total soluble protein under low N supply may be associated with the nitrogen metabolism due to an increase in transcripts levels encoding various proteins [68] or due to high protease activity that can lead to higher protein degradation [69]. It was also reported that high NUtE is linked with the acceleration of N-related physiological and biochemical processes [45]. Thus, increase in GS activity, as well as root total soluble protein under low nitrogen may be an adaptive strategy, helping the cotton plant in promoting better root systems for enhancing nitrogen uptake, assimilation, and finally NUE. These results suggest that NUE in cotton genotypes is likely determined by biochemical processes; thus, improving N metabolism through genetic approaches might improve NUE.

## 4. Materials and Methods

### 4.1. Plant Cultivation and Nitrogen Treatment

A greenhouse experiment was carried out at the Cotton Research Institute of the Chinese Academy of Agriculture Sciences, Anyang, China. The representative four cotton genotypes comprising of two N-efficient (CCRI-69 and Z-1017) and two N-inefficient (GD-89 and XLZ-30) genotypes were selected from the previously screened genotypes on the basis of dry biomass and NUE [31]. Healthy seeds of each genotype were germinated in a growth chamber. Seven-day-old seedlings with uniform size were transplanted into plastic containers (7 L) in the growth chamber (16/8 h light/dark cycle, 28 °C temperature, 60% relative humidity). During the first week, the seedlings were provided with half-strength Hoagland solution, followed by full-strength according to Iqbal et al. [39]. At two leaf-stage, seedlings were exposed to various N supplies 0.25, 0.5, 1, 2, 4, and 6 mM as Ca(NO_3_)_2_. A total concentration of 0–4 mM CaCl_2_ was added to control treatment to equalize calcium concentration among the treatments [75]. The solutions were replaced on a weekly basis and aerated with an electric pump. The position of each plant was randomly changed every week to eliminate the effect of position.

### 4.2. Plant Morphological Characteristics

Before harvest, the shoot length of the four randomly selected plants from each replication was measured with the help of a ruler [76]. The leaf area of each representative plant was recorded as described by Iqbal et al. [39]. After harvesting, the plants were placed in the oven at 105 °C for 1 h, followed by 80 °C for 48 h. The complete dried root, shoot, and total plant dry matter was measured with the help of an electronic balance (Shimadzu electronic balance, Tatzendpromenade 2, 07745 Jena, Germany).

### 4.3. Measurement of Photosynthetic Characteristics

Before harvest, photosynthetic measurement was conducted from the top three leaves developed after N treatments. The photosynthetic traits such as net photosynthetic rate, stomatal conductance, transpiration rate, and intercellular CO_2_ concentration were measured with the help of a portable photosynthesis system (Li-Cor-6400; Li-Cor, Inc., Lincoln, NE, USA) from 9:00 a.m. to 11:00 a.m. in the growth chamber, according to a previously reported method [77]. Carbon dioxide concentration inside the chamber was maintained at 400 ± 1 µmoL CO_2_ (mol air)^−1^, and the light intensity was set as 1000 μmol photon m^−2^ s^−1^, as recommended by Cao et al. [78].

### 4.4. Measurement of N Concentration and NUE Traits

Tissue N concentrations were measured through the Kjeldahl method [39]. The grounded samples of about 0.2 g were weighed and digested with H_2_SO_4_-H_2_O_2_ for N concentration analysis [79] using Bran + Luebbe Continuous-Flow AutoAnalyzer III (AA3-Australlia). Shoot dry matter data of the cotton genotypes in response to N supply were described by non-linear regression analysis, where α shows the maximum shoot dry matter and β represents the N supply at half maximum shoot dry matter or the rate of N at which α was reached (Table 2). The performance of genotypes was analyzed by directly comparing the values of α and β in a way that was similar to the well-known Michaelis–Menten enzyme kinetics Vmax and Km [53]. Additionally, NUE definitions for cotton genotypes in response to various N supply were calculated as follows:

(1) Total N accumulation (TNA) calculated as the N concentration x total plant dry weight (mg N) [80];
(2) Nitrogen utilization efficiency (NUtE) calculated as the total plant dry weight divided by N concentration (g^2^ TDW mg^−1^ N) [81];
(3) Nitrogen uptake efficiency (NUpE) calculated as TNA divided by root dry weight (mg N g^−1^ root dry matter) [82].

### 4.5. Measurement of N-Metabolizing Enzymatic Activities

NR activity was analyzed according to Silveira et al. [83] and expressed as microgram nitrogen dioxide per hour per gram. About 0.2 g of fresh samples were grounded in liquid nitrogen. Following the addition of fresh tissue sample (0.2 g) added with 2 mL extraction solution 2.0 mL extraction (25.0 mM phosphate buffer (pH 7.5), 5.0 mM cysteine, and Ethylene diamine tetra acetic acid (EDTA-Na_2_) and centrifuged at 8000 rpm for 10 min at 4 °C. From each sample, 0.4 mL supernatant was mixed with 1.6 mL of a mixture containing 1.2 mL of 0.1 M KNO_3_ phosphate buffer and 0.4 mL of 2.0 mg·mL^−1^ nicotinamide adenine dinucleotide (NADH) solution, while the control was kept as NADH-free. Both control and treatment samples were kept in a water bath at 30 °C for 30 min. Then, 1.0 mL of 1% p-aminobenzene sulfonic acid and 0.2% α-naphthylamine were added to the supernatant, color developed for 20 min, and centrifuged for 5 min at 4000 rpm. The absorbance was determined by calorimetry at a wavelength of 540 nm using a spectrophotometer (722N, Shanghai Metash Instrument Co., Shanghai, China).

GS activity was determined according to the previously established method [84]. The enzyme activity was assessed by grinding fresh samples (0.2 g) on ice with an extraction containing 2.0 mL of an extract (0.05 M Tris–HCl buffer, pH 8.0, 2.0 mM MgSO4, 2.0 mM dithiothreitol (DTT), and 0.4 M sucrose), and centrifuged at 15,000 rpm for 20 min at 4 °C. After centrifugation, 0.7 mL of supernatant was mixed with 1.6 mL of 0.1 M Tris–HCl buffer (pH 7.4, 80.0 mM MgSO_4_, 20.0 mM sodium glutamate, 20.0 mM cysteine, 2.0 mM EDTA, containing 80.0 mM HONH_3_Cl) and 0.7 mL of 40.0 mM ATP solution. The mixture was placed in a water bath at 25 °C for 30 min, to which 1.0 mL of a chromogenic reagent (0.2 M trichloroacetic acid, 0.37 M FeCl_3_, and 0.6 M HCl) was added, incubated for 15 min, and centrifuged at 5000 rpm for 10 min at 25 °C; then, the supernatant was collected and the absorbance was measured at a wavelength of 540 nm. The reaction mixture of 1.6 mL of 0.1 M Tris–HCl solution (pH 7.4, not containing 80.0 mM HONH_3_Cl) was added as control. GOGAT and GDH activities of both tissues were measured according to the previous method at a wavelength of 340 nm using a spectrophotometer [85]. The enzyme extraction from the samples were the same as those of GS. Then, 100 mM K+ phosphate (pH 7.6), containing 0.1% (*v/v*) 2-mercaptoethanol, was used to determine the activity of the GDH and GOGAT enzymes. In addition, the reaction solution of GOGAT was 2.5 mM α-ketoglutarate, 100 μM NADH, 10.0 mM L-glutamine, and 1.0 mM aminooxyacetate, and that of GDH was 2.5 mM α-ketoglutarate, 100.0 μM NADH, and 100.0 mM (NH_4_)_2_SO_4_. One unit of both GOGAT and GDH was calculated as the oxidation of 1 nmol of NADH per minute. 

### 4.6. Measurement of Total Soluble Protein and Total Free Amino Acids

In the current experiment, total soluble protein was also measured according to the supposed method by Bradford [86] using Coomassie Brilliant Blue (G-250) as a dye and albumin as standard according to [87]. Root and shoot samples (0.5 g) were grounded in liquid nitrogen and homogenized in phosphate buffer (5 mL). The samples were then placed in a water bath 100 °C (10 min) and then centrifuged at 3000× *g* for 5 min at 22–25 °C. Reaction mixture composed of 2 mL distilled Water (dH_2_O), enzyme extract (20 µL), and Bradford reagent (0.5 mL). Finally, the values were recorded at a 595 nm wavelength using distilled water as a blank control and bovine albumin (BSA) as a standard with the help of a spectrophotometer (UV-2600) (722N, Shanghai Metash Instrument Co., Shanghai, China).

For the determination of total free amino acids, the ninhydrin method was used as previously described [88] with some modifications [89]. Extraction buffer composed of acetic acid/sodium acetate (pH 5.4) and the final values of free amino acids were detected at 580 nm by using a spectrophotometer (UV-2600) (722N, Shanghai Metash Instrument Co., Shanghai, China).

### 4.7. Statistical Analysis

The effects of genotypes, N supply, and their interaction were evaluated by two-way ANOVA with a split-plot arrangement using Statistix 10 software (Analytical Software, Tallahassee, FL, USA). Nitrogen treatments were used as the main plot, whereas genotypes were used as subplot factor during analysis. Means were separated using the least significant test (LSD) at a 5% level of significance. Graphs were generated using GraphPad Prism 7, and PCA was calculated in OriginPro 2015 (b9.2.214, OriginLab Corporation, Northampton, MA, USA). Various studied traits were used to calculate the relationships with NUpE and NUtE using R package GeneNT [90], and the results were visualized according to the method of Iqbal et al. [91].

## 5. Conclusions

In conclusion, two NUE-contrasting genotypes, CCRI-69 and XLZ-30, were selected as N-efficient and N-inefficient genotypes, respectively. Of note, CCRI-69 showed higher NUE-related traits values, such as shoot dry matter, NUpE, NUtE, and N metabolism. Moreover, root-soluble protein and shoot glutamine synthetase were positively correlated with NUtE, whereas N-metabolizing enzymes and N concentration was positively correlated with NUpE. Interestingly, shoot glutamine synthetase shared more genotypic variation, suggesting the importance of glutamine synthetase in improving NUE. Moreover, enhanced accumulation root total soluble protein and shoot glutamine synthetase activity requires deep interpretation on the molecular level to fully understand the underlying mechanism of NUE.

## Figures and Tables

**Table 1:** Shoot length (cm), total dry matter (g plant^−1^), single leaf area (cm^2^), net photosynthetic rate (µmol m^−2^ s^−1^), stomatal conductance (mmol H_2_O m^−2^ s^−1^), transpiration rate (mmol m^−2^ s^−1^), and intercellular CO_2_ (mmol CO_2_ moL^−^¹ air) of contrasting nitrogen-efficient cotton genotypes under various nitrogen supplies (0.25, 0.5, 1, 2, 4, and 6 mM).

N Supply (mM)	Genotype	SL	TDM	SLA	Pn	gs	E	Ci
0.25	GD-89	5.95	1.40	16.02	6.40	0.274	3.31	324
	XLZ-30	5.68	1.34	13.71	5.23	0.263	2.93	311
	CCRI-69	10.45	1.73	23.43	6.73	0.279	3.21	251
	Z-1017	8.25	1.61	20.76	6.54	0.276	3.36	315
0.5	GD-89	10.78	1.63	29.99	6.70	0.284	3.04	267
	XLZ-30	9.95	1.49	27.94	5.67	0.282	3.33	294
	CCRI-69	16.25	1.96	47.71	7.09	0.298	4.22	259
	Z-1017	14.12	1.82	40.93	6.78	0.293	3.40	308
1	GD-89	16.38	2.16	45.80	7.47	0.291	3.19	260
	XLZ-30	13.08	1.98	39.69	6.51	0.280	3.46	283
	CCRI-69	18.45	2.70	69.00	7.84	0.305	3.80	232
	Z-1017	17.18	2.38	55.05	7.65	0.298	3.69	223
2	GD-89	19.38	2.34	56.65	7.77	0.290	3.98	269
	XLZ-30	16.58	2.27	48.45	7.18	0.281	3.39	290
	CCRI-69	22.38	3.06	79.36	8.46	0.316	4.06	212
	Z-1017	21.92	2.62	70.67	7.91	0.305	3.66	234
4	GD-89	20.62	2.47	79.72	8.34	0.318	3.84	219
	XLZ-30	18.15	2.38	57.69	7.65	0.290	3.37	235
	CCRI-69	26.22	3.18	104.32	8.94	0.322	4.30	224
	Z-1017	25.75	2.87	91.95	8.50	0.322	3.80	286
6	GD-89	22.78	2.47	96.50	9.03	0.279	4.18	226
	XLZ-30	19.85	2.35	78.85	8.48	0.291	3.67	220
	CCRI-69	26.52	3.30	120.65	9.99	0.301	4.47	209
	Z-1017	23.95	2.78	104.63	9.35	0.286	4.60	166
	N × G	**	***	***	ns	ns	ns	*
	CV-I (%)	11.88	5.04	21.42	10.33	3.66	10.25	12.8
	CV-II (%)	6.51	13.58	11.84	3.63	4.12	11.02	11.3

Note: ns, not significant; * *p* < 0.05, ** *p* < 0.01, *** *p* < 0. SL, shoot length; TDM, total plant dry matter; SLA, single leaf area; Pn, photosynthetic rate; gs, stomatal conductance; E, transpiration rate; Ci, intercellular CO_2_ concentration; CV-I, coefficient of variation (main plot); CV-II, coefficient variation (subplot).

**Table 2:** Kinetic parameters for cotton genotypes of the shoot dry-matter (α, g plant^−1^) and N supply response (β mM), where *R^2^* or coefficient of determination was *p* < 0.05 for all genotypes.

Genotypes	α	β	R^2^
CCRI-69	2.79 a	0.24 c	0.64
Z-1017	2.50 ab	0.33 b	0.63
GD-89	2.16 b	0.40 b	0.60
XLZ-30	1.99 b	0.59 a	0.20

Note: means followed by the same letters within the same columns are not different statistically at 5% probability using least significant test (LSD).

## Supplementary Materials

The following are available online at https://www.mdpi.com/2223-7747/9/2/250/s1: Table S1: Principal component analysis (PCA) of morphophysiological and biochemical traits of contrasting N-efficient cotton genotypes under various nitrogen supply (0.25, 0.5, 1, 2, 4 and 6 mM).
