# Supplementary material for: Variations in Nitrogen Metabolism are Closely Linked with Nitrogen Uptake and Utilization Efficiency in Cotton Genotypes under Various Nitrogen Supplies
Source: Plants (Basel). 2020 Feb 15;9(2):250. doi: 10.3390/plants9020250 (PMC7076418; doi:10.3390/plants9020250)
Supplement: Supplementary file 1 [file plants-09-00250-s001.pdf]

Supplementary Table S1. Principal component analysis (PCA) of morphophysiological and biochemical traits of contrasting N efficient cotton genotypes under various nitrogen supply (0.25, 0.5, 1, 2, 4 and 6 mM).

Traits	PC1	PC2
Shoot length	0.22	0.05
Single leaf area	0.23	-0.03
Total plant dry matter	0.22	0.19
Photosynthetic rate	0.22	0.00
Stomatal conductance	0.15	0.35
Intercellular CO_2 concentration	-0.18	-0.01
Transpiration rate	0.20	0.12
Shoot nitrogen concentration	0.22	-0.14
Root nitrogen concentration	0.22	-0.18
Nitrogen utilization efficiency	-0.11	0.68
Nitrogen uptake Efficiency	0.22	-0.11
Shoot nitrate reductase activity	0.22	0.04
Root nitrate reductase activity	0.22	0.09
Shoot glutamine synthetase activity	0.20	0.24
Root glutamine synthetase activity	0.22	0.03
Shoot glutamate synthase activity	0.21	0.09
Root glutamate synthase activity	0.21	0.16
Shoot glutamate dehydrogenase activity	0.22	0.02
Root glutamate dehydrogenase activity	0.22	0.03
Shoot free amino acid	0.22	-0.02
Root free amino acid	0.21	-0.15
Shoot total soluble protein	0.23	0.03
Root total soluble protein	-0.17	0.42
Eigen value	19.32	1.43
Variance contribution rate (%)	84.01	6.22
Cumulative percentage (%)	84.01	90.23
